# Late Onset Descemet Membrane Detachment after Radial Keratotomy Resolved with Medical Therapy

**DOI:** 10.1155/2017/5804965

**Published:** 2017-10-31

**Authors:** P. Rosetta, E. F. Legrottaglie, R. Vinciguerra, P. Vinciguerra

**Affiliations:** ^1^Eye Center, Humanitas University, Humanitas Clinical and Research Hospital, Rozzano, Italy; ^2^St. Paul's Eye Unit, Royal Liverpool University Hospital, Liverpool, UK

## Abstract

**Purpose:**

To report a case of a Descemet membrane's (DM) detachment after radial keratotomy (RK).

**Methods:**

A patient (male) underwent RK (16 cuts) 20 years before referring to the Eye Center of Humanitas (Milan) for a progressive visual loss. The slit-lamp examination showed severe corneal stromal edema and a large DM detachment in the lower half of the cornea. Anterior segment optical coherence tomography (AS-OCT) and endothelial cells count confirmed DM detachment and endothelial cells damage. Descemet Stripping Automated Endothelial Keratoplasty (DSAEK) was planned and topical hypertonic therapy was prescribed before the surgery.

**Results:**

Eight months later, the patient mentioned a spontaneous increase in visual acuity; the slit-lamp examination and the AS-OCT displayed a recovery of corneal transparency with a resolution of DM detachment.

**Conclusions:**

This is the first report of spontaneous DM detachment with severe corneal edema after RK. We suggest that hypertonic therapy may reduce DM detachment and restore corneal transparency.

## 1. Introduction

Radial keratotomy (RK) is an old procedure invented in 1974 by Svyatoslav Fyodorov aimed to correct myopia and corneal astigmatism [1]. However, RKs are well known to cause many complications such as corneal perforations [2], decentration [3], over- or undercorrection [2], astigmatism, contact lens intolerance [3], stromal melting, endothelial cell loss [4], and infectious keratitis [5]. In more detail, endothelial cell loss has been described and correlated with small optical zone and perforations [4]. The aim of our article is to report a case of a Descemet membrane's (DM) detachment after radial keratotomy (RK) resolved without surgical intervention.

## 2. Case Report

**Figure 1:**
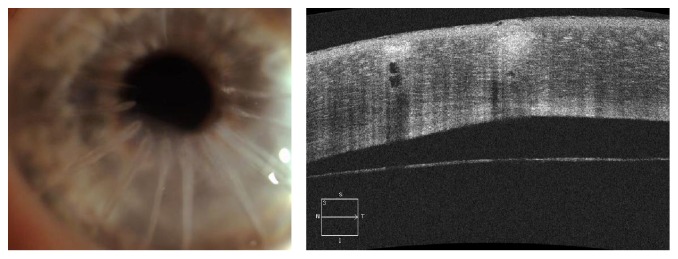
DM detachment.

In 1992 a young man (43 years old) underwent bilateral radial keratotomy, performed with 16 corneal cuts, to correct high myopia and corneal astigmatism; the surgery and postoperative period passed without any adverse events. 20 years after surgery, this patient came to the corneal service of the Eye Center of Humanitas Clinical and Research Institute of Rozzano (Milan) to perform a complete ophthalmological examination in order to understand the cause of his progressive visual loss from which he had been suffering since about six months. He underwent a complete ophthalmological evaluation, a corneal topography (Costruzione Strumenti Oftalmici [CSO], Florence, Italy), a Pentacam tomography (Oculus Inc., Lynnwood, WA), an anterior optical coherence tomography AS-OCT (Cirrus, Carl Zeiss Meditec AG, Germany), and endothelial cell count (Konan Medical Inc., Hyogo, Japan). The AS-OCT showed a DM detachment and an extensive corneal edema of the lower half of the cornea was identified as the main cause of visual decrease (Figure 1). His best corrected visual acuity (BCVA) was 6/20 with −3.00 sphere and −4.50 cylinder axis 90°. Corneal pachymetry was 950 microns with the thinnest point of 361 microns and thickness in the pupil center of 891 microns. The endothelial cell count was about 427 cells/mm^2^.

**Figure 2:**
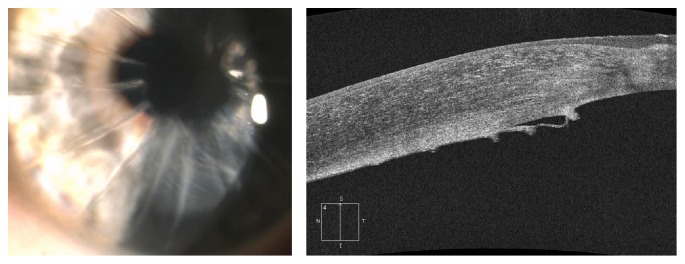
Descemet membrane folds.

The possible therapeutic solutions we hypothesized were rebubbling (introduction of an air bubble in the anterior chamber), a DSAEK, or PK (Penetrating Keratoplasty). In order to preoperatively reduce the corneal edema, a topical hypertonic solution (sodium chloride 5%—1878 mOsm/l) was prescribed, one drop four times a day, before surgery until the preoperative visit. In the preoperative visit, 8 months later, the patient mentioned a partial improvement of visual acuity; the AS-OCT images showed no stromal dialysis, while the slit-lamp examination revealed a corneal transparency and significant reduction of corneal edema (Figure 2). BCSVA was 10/20 with +2.00 sph and −1.00 cyl 120 and central pachymetry measured 630 *μ*m.

## 3. Discussion and Conclusion

In literature there is another case report of bullous keratopathy after RK [6]. In that study it was postulated that the corneal damage, due to endothelial decompensation many years after RK, was caused by the long term damage arising from the corneal incisions (Sato's anterior-posterior radial keratotomy) in combination with endothelial losses due to aging [6].

In the study of Mac Rae [7], radial keratotomy does not cause accelerated endothelial cell loss after a 4- to 10-year period. In particular, after a follow-up over a 7-year period, the rate of endothelial cell loss decreased from 3.3% 1 year after RK to 0.4% every following year. Although other studies showed a trend of stabilization in the endothelial cell count [4, 8], the long term RK effects on the endothelium 20 to 30 years after surgery are unknown. In our experience, a severe corneal decompensation many years later may appear as a result of Descemet membrane detachment originated under the corneal dialysis through incisions and caused by an osmotic flow and later extended without endothelial losses. The comprehension of edema pathogenesis is always a priority for the therapy planning. The topical hypertonic solution, inverting the osmotic flow, induced a progressive absorption of the fluid between the Descemet membrane and the stroma and produced the attachment of the two layers. The result was a huge reduction of the corneal edema and an improvement of the visual acuity.

In literature there are many cases of DM detachments after intraocular surgery [9–11] with spontaneous resolution in case of small DM detachments, but this evidence is not frequent when there is a large DM detachment.

Also in our case report the patient complained of visual decrease for about 6 months before the visit at the cornea center of Humanitas Research Hospital, because of an extensive DM detachment of half of the cornea, without spontaneous resolution, occurring 20 years after the corneal surgery.

Only the introduction of the hypertonic solution therapy induced the improvement of visual acuity, so we hypothesize the crucial influence of pharmacological therapy to restore corneal transparency. Furthermore, in literature there are four more cases described of Descemet Stripping Automated Endothelial Keratoplasty (DSAEK) due to bullous keratopathy after anterior-posterior radial keratotomy (APRK) as an effective surgical option [12]. We agree with Nakatani and Murakami about the choice of DSAEK as the preferable less invasive surgical approach in the bullous keratopathy after APRK, but our clinical experience confirms the indication to a pharmacological, noninvasive treatment before any surgery.
